# Dynamic prediction of kidney allograft and patient survival using post-transplant estimated glomerular filtration rate trajectory

**DOI:** 10.1093/ckj/sfae314

**Published:** 2024-10-16

**Authors:** Khandoker Shuvo Bakar, Armando Teixeira-Pinto, Ryan Gately, Farzaneh Boroumand, Wai H Lim, Germaine Wong

**Affiliations:** Sydney School of Public Health, University of Sydney, Sydney, Australia; Sydney School of Public Health, University of Sydney, Sydney, Australia; Centre for Kidney Research, Kids Research Institute, The Children's Hospital at Westmead, Sydney, Australia; Department of Kidney and Transplant Services, Princess Alexandra Hospital, Brisbane, Australia; Sydney School of Public Health, University of Sydney, Sydney, Australia; Department of Renal Medicine, Sir Charles Gairdner Hospital, Perth, Australia; Medical School, University of Western Australia, Perth, Australia; School of Medical & Health Sciences, Edith Cowan University, Perth, Australia; Sydney School of Public Health, University of Sydney, Sydney, Australia; Centre for Kidney Research, Kids Research Institute, The Children's Hospital at Westmead, Sydney, Australia; Department of Renal Medicine, Westmead Hospital, Sydney, Australia

**Keywords:** allograft loss, death, dynamic modeling, eGFR trajectory, kidney transplant

## Abstract

**Background:**

Allograft loss is the most feared outcome of kidney transplant recipients. We aimed to develop a dynamic Bayesian model using estimated glomerular filtration rate (eGFR) trajectories to predict long-term allograft and patient survivals.

**Methods:**

We used data from the Australian and New Zealand Dialysis and Transplant registry and included all adult kidney transplant recipients (1980–2017) in Australia (derivation cohort) and New Zealand (NZ, validation cohort). Using a joint model, the temporal changes of eGFR trajectories were used to predict patient and allograft survivals.

**Results:**

The cohort composed of 14 915 kidney transplant recipients [12 777 (86%) from Australia and 2138 (14%) from NZ] who were followed for a median of 8.9 years. In the derivation cohort, eGFR trajectory was inversely associated with allograft loss [every 10 ml/min/1.73 m^2^ reduction in eGFR, adjusted hazard ratio [HR, 95% credible intervals (95%CI) 1.31 (1.23–1.39)] and death [1.12 (1.10–1.14)]. Similar estimates were observed in the validation cohort. The respective dynamic area under curve (AUC) (95%CI) estimates for predicting allograft loss at 5-years post-transplantation were 0.83 (0.75–0.91) and 0.81 (0.68–0.93) for the derivation and validation cohorts.

**Conclusion:**

This straightforward model, using a single metric of eGFR trajectory, shows good model performance, and effectively distinguish transplant recipients who are at risk of death and allograft loss from those who are not. This simple bedside tool may facilitate early identification of individuals at risk of allograft loss and death.

KEY LEARNING POINTS **What was known**:

- Single time-point eGFR is associated with patient important outcomes such as death and allograft loss in kidney transplant recipients.
- eGFR trajectory is associated with progression to kidney failure and death in the general population, but a consistent relationship between eGFR trajectory and allograft loss and death after kidney transplantation remains poorly defined.

**This study adds**:

- The single metric of eGFR trajectory, along with other readily available donor, recipient, and transplant-related characteristics can reliably predict the risk of allograft loss and death in kidney transplant recipients, with improvements in predictive accuracy with increasing number of post-transplant eGFR timepoints.
- An average AUC of 0.8 reflects the model's strong ability to identify kidney transplant recipients at risk of graft loss and death within 5 years of initial risk assessments.

**Potential impact**:

- This easy-to-use dynamic eGFR risk calculator, developed using a Shiny Application and derived from a large cohort of kidney transplant recipients in Australia and New Zealand, may facilitate early identification of individuals at risk of allograft loss and death. This tool may assist in guiding interventions aimed at potentially slowing the progression of disease processes.

## INTRODUCTION

Over the past three decades, the kidney transplant community has seen a significant improvement in short- and medium-term allograft outcomes, with observed 5-year allograft survival exceeding 90% in many countries [1–4]. Although there have been some improvements in longer-term allograft outcomes, progress has been slow. The median allograft survival for primary deceased donor kidney transplant in the USA is ∼15 years, with similar estimates observed in Europe and Australia [2, 3]. Optimization of allograft function is therefore one of the most important outcomes identified by patients, caregivers, and health professionals in kidney transplantation [5–7], because poor allograft outcome and return to dialysis after allograft loss are major risk factors of premature death [8, 9]. Multiple pre-transplant clinical and immunological factors, including donor and recipient characteristics, may contribute to late kidney allograft loss, but a simple, globally accepted metric to reliably predict long-term allograft survival remains elusive.

Recent research has used the Bayesian joint modeling approach to capture repeated temporal measurements of post-transplant estimated glomerular filtration rate (eGFR) for the prediction of long-term allograft survivals, but not patient survival [10, 11]. The predictive performance of these models improved as eGFR values were added to the algorithm over the life-course of the kidney transplant, with the time varying area under the receiver operating characteristic curve (AUC) increasing from 0.7 in year 1 to 0.87 in year 5 post-transplant. In addition to eGFR measurements, these models also included proteinuria, immunological factors such as *de novo* donor-specific human leukocyte antigen (HLA) antibody, and detailed allograft biopsy information. However, some of these data are not readily available in many settings or are not collected routinely, such as in low-health resource settings. Therefore, having a prognostic tool that uses simple measures of kidney function such as eGFR, to identify those at the highest risk of rapid deterioration of kidney allograft function can allow early intervention, such as modification of immunosuppression, to prevent allograft loss and subsequent death. In this study, we developed and evaluated the performance characteristics of a dynamic Bayesian risk model that assessed the temporal relationship between eGFR trajectory, allograft, and patient survivals in a cohort of deceased and living donor kidney transplant recipients. The proposed model is also converted into a web-based application (web: https://centreforkidneyresearch.shinyapps.io/CKR_JMapp/) for easy access to clinicians and researchers.

## MATERIALS AND METHODS

### Study design

**Figure 1:**
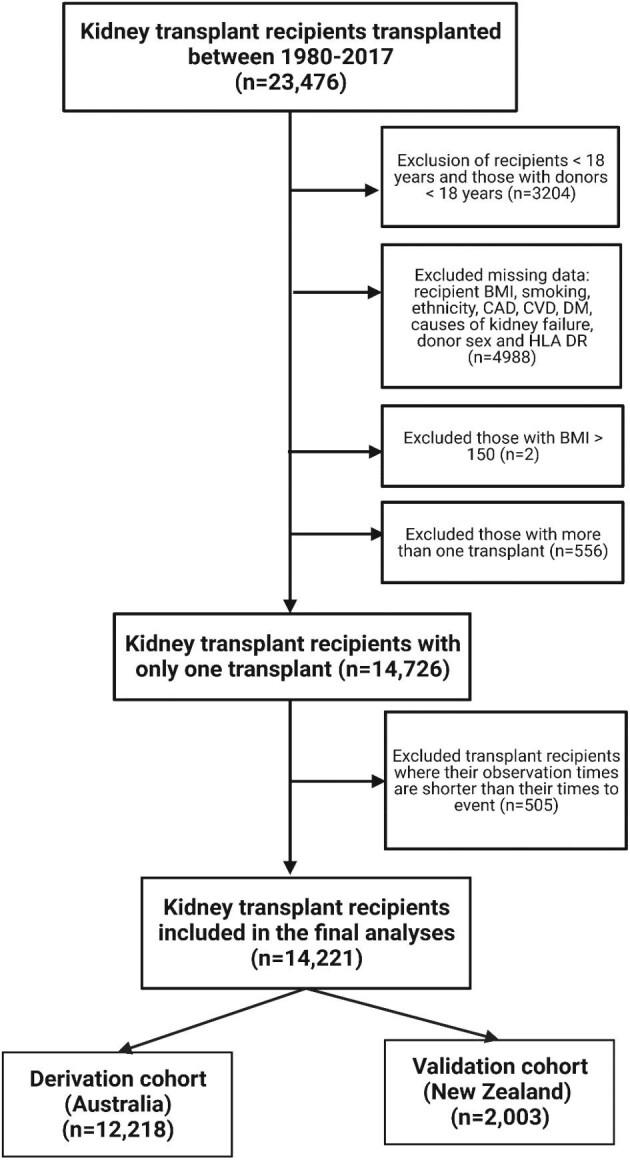
Flow diagram of the study cohort of adult kidney transplant recipients in Australia and New Zealand between 1980 and 2017.

Using data from the Australia and New Zealand Dialysis and Transplant (ANZDATA) registry, all adult patients (aged 18 years and over) with kidney failure in Australia and New Zealand, who have received first kidney transplants from live and deceased donors between 1980 and 2017 were included (Fig. 1). Multi-organ transplant recipients and those with previous kidney allografts were excluded from this analysis. The University of Western Australia Human Research Ethics Committee approved the conduct of this study (ethics reference number 2022/ET000697). The study was reported in accordance with the Transparent Reporting of a Multivariable Prediction Model for Individual Prognosis or Diagnosis (TRIPOD) guidelines [12].

### Recipient and donor characteristics

Data extracted from the registry included donor characteristics such as age, sex, and donor type (either living or deceased); recipient characteristics including age, sex, body mass index (BMI) at time of transplantation, smoking status, ethnicity, comorbidities at time of transplantation (including coronary artery disease, cerebrovascular disease, peripheral vascular disease, and diabetes), primary cause of kidney failure, and duration of dialysis; and transplant-related factors of the number of HLA-DR mismatches and era.

### Exposure

The main exposure was eGFR, calculated using the Chronic Kidney Disease Epidemiology Collaboration (CKD-EPI) equation [13]. eGFR measurements were available at prespecified time points of 3, 6, 12, 24, 36, and 60 months after transplantation from the ANZDATA registry.

### Outcome measures

The primary endpoint was overall allograft loss, defined as a return to dialysis or death with a functioning allograft. Recipients were censored at lost to follow-up, the end of their follow-up time, or 31 December 2017, whichever occurred first. The secondary endpoint was all-cause death. Patients who lost their allografts and returned to dialysis or had received a pre-emptive second transplant during the follow-up time were censored at the time of allograft loss.

### Statistical analysis

The baseline characteristics of the study cohort were reported using means [standard deviations (SD)], numbers (percentages) and medians [interquartile range (IQR)] according to the data distribution. The study population was divided into two groups; a derivation cohort that included kidney transplant recipients from Australia and a validation cohort that included recipients from New Zealand. Comparisons of baseline characteristics between the derivation and validation cohorts were made using chi-square test, *t*-test, and Mann–Whitney *U*-test, where appropriate.

We implemented the Bayesian joint modeling technique [14–16], where longitudinal and survival models were used to obtain the predictors of the primary outcome of the probability of allograft survival and the secondary outcome of patient survival. A linear mixed model (for the longitudinal measurement of eGFR) and multivariable Cox proportional hazard models were constructed for both allograft and patient survival for the survival analyses as a part of the Bayesian joint modeling approach [17]. In the Bayesian setup, we analyzed longitudinal and time-to-event data simultaneously with the goal of understanding the association between eGFR and primary (allograft survival) and secondary (patient survival) outcomes. To appropriately utilize the Bayesian joint model, we implemented prior sensitivity analyses, where initially vague or non-informative prior distributions were used for the model parameters by considering large variability. Consequently, we limited the variability and checked the performance of the Bayesian joint model using the log of the posterior predictive density [18, 19]. We observed that our model was less sensitive to the prior distribution (see the Supplementary section) and was mainly driven by the large amount of kidney transplant recipient data.

The clinical and immunological variables included in the multivariable models were selected *a priori*, based on previously established biological relationships with the outcome measures [20], and these variables are described in Table 1. In the joint model, we included the multivariable Cox model with a mixed model that estimated the trajectories of eGFR changes over time for the prediction of overall kidney allograft and patient survivals, whereas the longitudinal model was used to estimate the model parameters up to the follow-up period of the eGFR measurement. The dynamic trajectories of overall allograft and patient survivals for a new (or existing) patient over time were estimated based on temporal changes of eGFR measurement(s). The performance of the prediction model was based on the AUC and calibration [21]. The development model using the data from the derivation cohort (Australian recipients) was first validated internally using the bootstrapping method with subsamples from the Australian cohort. We used 1000 bootstrap replications for each of 10 000 Markov chain Monte Carlo posterior predictive samples and estimated the dynamic AUCs for different time thresholds considering the prediction horizon at the median follow-up time after first transplantation. Similar models were implemented and statistically validated for the validation cohort (New Zealand recipients). We used calibration plots between the predicted and the observed number of allograft and patient survivals. Using a standard calibration approach [22], we estimated the probabilities in deciles according to patient follow-up. We also used calibration statistics, Brier score, average absolute difference in predicted and loess-calibrated probabilities, and Spiegelhalter *Z*-test, to assess for calibration accuracy [23]. These calibration statistics were used to check the robustness and accuracy of the Bayesian joint model we developed for obtaining projections of overall allograft loss and patient death. We then performed dynamic predictions for both overall allograft and patient survivals for the validation cohorts using data from the derivation cohort. To perform this, we used 3-, 6-, 12-, 24-, 36-, and 60-month

eGFR measurements post-transplant to obtain dynamic predictions for overall allograft and patient survival curves for each of these time points. Analyses were undertaken using the R programming language, and statistical significance is measured using Bayesian credible intervals with projection validations statistics.

**Table 1:** Baseline characteristics of kidney transplant recipients transplanted between 1980 and 2017.

	Summary statistics		
Variables	Australia (*N* = 12 777)	New Zealand (*N* = 2138)	*P* value
eGFR (ml/min/1.73 m^2^, mean ± SD)	53.44 ± 19.78	52.95 ± 18.25	.2
**Patient characteristics**			
Age (year, mean ± SD)	48.1 ± 13.6	47.3 ± 13.9	<.001
Sex			.7
Female (*n*, %)	4 825 (37.8)	797 (37.3)	
Male (*n*, %)	7952 (62.2)	1341 (62.7)	
BMI (kg/m^2^, mean ± SD)	25.9 ± 5.3	26.3 ± 5.2	.002
Smoking history			.041
Never (*n*, %)	7204 (56.4)	1256 (58.7)	
Former/current (*n*, %)	5573 (43.6)	882 (41.3)	
Ethnicity			<.001
Caucasian (*n*, %)	10 461 (81.9)	1452 (67.9)	
Indigenous Australian (*n*, %)	450 (3.5)	1 (<0.1)	
New Zealand Māori (*n*, %)	76 (0.6)	294 (13.8)	
Others/not recorded (*n*, %)	1790 (14.0)	391 (18.3)	
Prior coronary artery disease			.6
No (*n*, %)	12 756 (84.2)	1881 (88)	
Yes (*n*, %)	1601 (12.5)	199 (9.3)	
Suspected (*n*, %)	420 (3.3)	58 (2.7)	
Prior cerebrovascular disease			.4
No (*n*, %)	12 146 (95.1)	2042 (95.5)	
Yes (*n*, %)	491 (3.8)	20 (3.6)	
Suspected (*n*, %)	140 (1.1)	20 (0.9)	
Diabetes mellitus			<.001
No (*n*, %)	10 213 (79.9)	1783 (83.4)	
Yes (*n*, %)	2564 (20.1)	355 (16.6)	
Primary causes of kidney failure			<.001
Hypertension/renovascular disease (*n*, %)	657 (5.1)	139 (6.5)	
Glomerulonephritis (*n*, %)	5572 (43.6)	964 (45.1)	
Diabetes (*n*, %)	1726 (13.5)	280 (13.1)	
Cystic (*n*, %)	1868 (14.6)	296 (13.8)	
Analgesic nephropathy (*n*, %)	145 (1.1)	7 (0.3)	
Others (*n*, %)	2809 (22)	452 (21.1)	
Dialysis duration (in years)	2.5 ± 2.5	2.5 ± 2.7	.001
**Donor characteristics**			
Age (years, mean ± SD)	46.7 ± 14.4	43.2 ± 13.0	<.001
Sex			<.001
Female (*n*, %)	6077 (47.6)	1124 (52.6)	
Male (*n*, %)	6700 (52.4)	1014 (47.4)	
Donor type			<.001
Deceased donor (*n*, %)	8531 (66.8)	1152 (53.9)	
Living donor (*n*, %)	4246 (33.2)	986 (46.1)	
**Transplant-related characteristics**			
HLA-DR mismatches			<.001
0 (*n*, %)	3953 (30.9)	677 (31.7)	
1 (*n*, %)	5177 (40.5)	1125 (52.6)	
2 (*n*, %)	3647 (28.5)	336 (15.7)	
Transplant era			<.001
1980–1990 (*n*, %)	394 (3.1)	22 (1)	
1990–2000 (*n*, %)	2116 (16.5)	385 (18)	
2000–2010 (n, %)	3858 (30.2)	698 (32.7)	
2010–2017 (*n*, %)	6422 (50.2)	1033 (48.3)	
Immunosuppression			
Prednisone	12 233 (95.7)	2125 (99.3)	
Calcineurin inhibitors			
Cyclosporin A	5293(41.4)	1688 (78.9)	
Tacrolimus	6724 (52.6)	395 (18.5)	
None	760 (5.9)	55 (2.6)	
Anti-metabolities			
Azathioprine	1899 (14.9)	377 (17.6)	
Mycophenolate mofetil	8998 (70.4)	1266 (59.2)	
None	1880 (14.7)	495 (23.2)	

Data expressed as number (percentage) or mean (SD).

## RESULTS

### Study cohort

The study cohort composed of 14 915 kidney transplant recipients, including 12 777 (86%) recipients from Australia (derivation cohort) and 2138 (14%) recipients from New Zealand (validation cohort). The mean eGFR (SD) for the study cohort was 53.4 (19.8) ml/min/1.73 m^2^. The mean age (SD) of the study cohort was 48 (13.7) years, with 38% females. Most of the cohort were of European ethnic backgrounds (80%). Approximately 12% of the participants had prevalent coronary artery disease, 4% had cerebrovascular disease, and 20% had diabetes at the time of transplantation. The most common primary causes of kidney failure were glomerulonephritis (44%), followed by diabetic kidney disease (13%) and cystic disease (15%). The mean age of the donor (SD) was 46.2 (14.3) years and 48% of the donors were women. Among donors, 65% and 35% were deceased and live donors, respectively. Most of the data (50%) in the study cohort were collected between 2010 and 2017, while only 3% of the data was from the previous decade between 1980 and 1990.

### Baseline characteristics of the derivation (Australian) and validation (New Zealand) cohorts

The mean age (SD) of kidney transplant recipients in Australia (derivation cohort) was 48.1 (13.6) years and the mean BMI (SD) at the time of transplant of 25.9 (5.3) kg/m^2^. Almost 38% of the recipients were female in the derivation cohort and <4% were Indigenous Australians. The average duration of dialysis (SD) was 2.6 (2.5) years. The mean age of the donor (SD) was 46.7 (14.4) years and 48% of the donors were women. Almost 70% of the donors were of deceased donor type. The mean age (SD) of transplant recipients in New Zealand (validation cohort) was 47.3 (13.9) years with a mean BMI (SD) of 26.3 (5.2) kg/m^2^. Similar to the derivation cohort, 63% of the recipients were men and 14% were New Zealand Māori. The mean (SD) years on dialysis prior to transplantation for the New Zealand cohort was 2.5 (2.7) years. Of all donors in New Zealand, ∼53% were men and 54% were deceased donors. The mean age of the donor (SD) was 43.2 (13) years. Compared to the validation cohort, the mean eGFR (SD) for the derivation cohort was higher at baseline, recipients were also older, more likely to have diabetes mellitus and prevalent vascular disease, and more likely to have received kidney transplants with one or more HLA-DR mismatches.

### Allograft and patient survivals in the derivation and validation cohorts.

Over a median (IQR) patient follow-up period of 8.9 (IQR 4.7–14.6) years, including 4.6 (IQR 1.6–9.9) years of allograft follow-up, 38% of recipients experienced allograft loss and 25% died. The median (IQR) patient follow-up time for the Australian derivation and NZ validation cohorts were 8.9 (IQR 4.8–14.7) and 8.8 (IQR 4.3–14.3) years, respectively. The median (IQR) allograft survival time (in years) after transplantation for the derivation and validation cohorts were 7.6 (IQR 3.7–12.2) and 6.9 (IQR 3.6–10.8), respectively. The overall median patient survival time (in years) after transplantation was 8.7 (IQR 4.6–13.9) years for the derivation cohort and 8.7 (IQR 4.6–12.9) for the validation cohort.

### Factors associated with a temporal reduction in eGFR post-transplant (Table 2)

Factors associated with a temporal reduction in eGFR included increasing recipient age, recipient male sex, Indigenous Australians and New Zealand Māori Peoples, prior smoking history, higher baseline BMI, increasing dialysis vintage prior to transplantation, and increasing donor age. On average, the eGFR was 2.82 ml/min/1.73 m^2^ lower for men compared to women.

**Table 2:** Factors associated with the longitudinal change in the mean eGFR over time (multivariable analysis).

	Change in mean eGFR (95% credible interval)
**Patient characteristics**	
Age (per year increase)	−0.25 (−0.37, −0.13)
Sex	
Female	
Male	−2.82 (−4.47, −1.09)
BMI (per kg/m^2^ increase)	−0.51 (−0.55, −0.44)
Smoking history	
Never	
Former/current	−1.45 (−2.04, −0.94)
Ethnicity	
Caucasian	
Indigenous Australian	−2.24 (−3.73, −0.48)
New Zealand Māori	−4.68 (−8.35, −0.96)
Others/not recorded	2.31 (1.48, 3.12)
Prior coronary artery disease	
No	
Yes	0.71 (−0.22, 1.50)
Suspected	0.49 (−0.83, 1.81)
Prior cerebrovascular disease	
No	
Yes	1.14 (−0.01, 2.23)
Suspected	1.14 (−0.62, 3.73)
Diabetes mellitus	
No	
Yes	1.81 (0.69, 3.63)
Primary cause of kidney failure	
Hypertension/renovascular disease	
Glomerulonephritis	−1.52 (−2.65, −0.40)
Diabetes	−0.57 (−2.34, 0.85)
Cystic	−0.59 (−1.70, 0.80)
Analgesic nephropathy	−0.03 (−2.85, 2.92)
Others	−1.54 (−2.77, −0.60)
Dialysis duration (per year increase)	−0.34 (−0.49, −0.22)
**Donor characteristics**	
Age (years)	−0.55 (−0.57, −0.54)
Sex	
Female	
Male	2.93 (2.33, 3.53)
Donor type	
Deceased donor	
Living donor	4.42 (3.89, 4.96)
**Transplant-related characteristics**	
HLA-DR mismatches	
0	
1	−0.51 (−1.08, 0.06)
2	−1.08 (−1.60, −0.53)
Transplant era	
1980–1990	
1990–2000	4.13 (−1.14, 8.42)
2000–2010	10.88 (6.81, 15.79)
2010–2017	15.94 (11.18, 20.51)

Data expressed as adjusted mean [and 95% credible interval (95%CI)] change in eGFR.

### Factors associated with allograft loss and patient death (Table 3)

Using data from the derivation cohort, an inverse association between eGFR trajectory and the risk of overall allograft loss was observed such that for every 10 ml/min/1.73 m^2^ reduction in eGFR over 5 years post-transplant, there was a 31% increased risk of overall allograft loss (adjusted HR 1.31, 95%CI 1.23–1.39). Similarly, we observed an inverse association between eGFR trajectory and the risk of all-cause death. For every 10 ml/min/1.73 m^2^ reduction in eGFR, there was a 12% (adjusted HR 1.12, 95%HR 1.10–1.14) increased risk of patient death over the 5-year period.

**Table 3:** Risk factors for allograft loss and patient death among kidney transplant recipients (*n* = 12 777).

	Allograft loss Adjusted HR (95% CI)	Patient death Adjusted HR (95% CI)
**Patient characteristics**		
Sex		
Female	1.00	1.00
Male	1.09 (1.01, 1.16)	1.02 (0.94, 1.10)
BMI (per kg/m^2^ increase)	1.01 (1.00, 1.02)	1.02 (1.02, 1.03)
Smoking history		
Never	1.00	1.00
Former/current	1.18 (1.11, 1.25)	1.34 (1.25, 1.42)
Ethnicity		
Caucasian	1.00	1.00
Aboriginal and Torres Strait Islander Peoples	1.95 (1.71, 2.25)	1.40 (1.20, 1.62)
New Zealand Māori	1.26 (0.89, 1.84)	0.67 (0.42, 1.07)
Others/not recorded	1.01 (0.90, 1.12)	0.79 (0.69, 0.91)
Prior coronary artery disease		
No	1.00	1.00
Yes	1.39 (1.28, 1.53)	1.91 (1.76, 2.09)
Suspected	1.30 (1.11, 1.51)	1.72 (1.46, 2.05)
Prior cerebrovascular disease		
No	1.00	1.00
Yes	1.45 (1.25, 1.65)	1.68 (1.45, 1.94)
Suspected	1.42 (1.03, 1.95)	1.43 (1.10, 1.84)
Diabetes		
No	1.00	1.00
Yes	1.37 (1.20, 1.57)	1.74 (1.50, 1.99)
Primary cause of kidney failure		
Hypertension/renovascular disease	1.00	1.00
Glomerulonephritis	0.82 (0.70, 0.95)	0.61 (0.53, 0.71)
Diabetes	0.94 (0.77, 1.12)	0.79 (0.66, 0.98)
Cystic	0.77 (0.65, 0.90)	0.83 (0.70, 0.98)
Analgesic nephropathy	1.57 (1.23, 2.03)	1.91 (1.53, 2.46)
Others	0.87 (0.76, 0.99)	0.64 (0.53, 0.75)
Dialysis duration (per year increase)	1.05 (1.04, 1.06)	1.08 (1.06, 1.10)
**Donor characteristics**		
Age (per year increase)	1.00 (1.00, 1.01)	1.01 (1.01, 1.01)
Sex		
Female	1.00	1.00
Male	0.99 (0.92, 1.06)	1.03 (0.97, 1.10)
Donor type		
Deceased donor	1.00	1.00
Living donor	0.83 (0.77, 0.89)	0.69 (0.64, 0.76)
**Transplant-related characteristics**		
HLA-DR mismatches		
0	1.00	1.00
1	1.16 (1.08, 1.26)	0.97 (0.89, 1.06)
2	1.25 (1.17, 1.33)	1.24 (1.12, 1.37)
Transplant era		
1980–1990	1.00	1.00
1990–2000	0.78 (0.68, 0.92)	1.58 (1.36, 1.85)
2000–2010	0.65 (0.57, 0.76)	1.33 (1.12, 1.58)
2010–2017	0.59 (0.52, 0.71)	1.16 (1.00, 1.38)
Predicted eGFR (per 10 ml/min/1.73 m^2^ increase)	1.31 (1.23, 1.39)	1.12 (1.10, 1.14)

Data expressed as adjusted median hazard ratio and 95% credible interval (HR, 95%CI).

### Performance characteristics of the dynamic joint model (derivation cohort)

**Figure 2:**
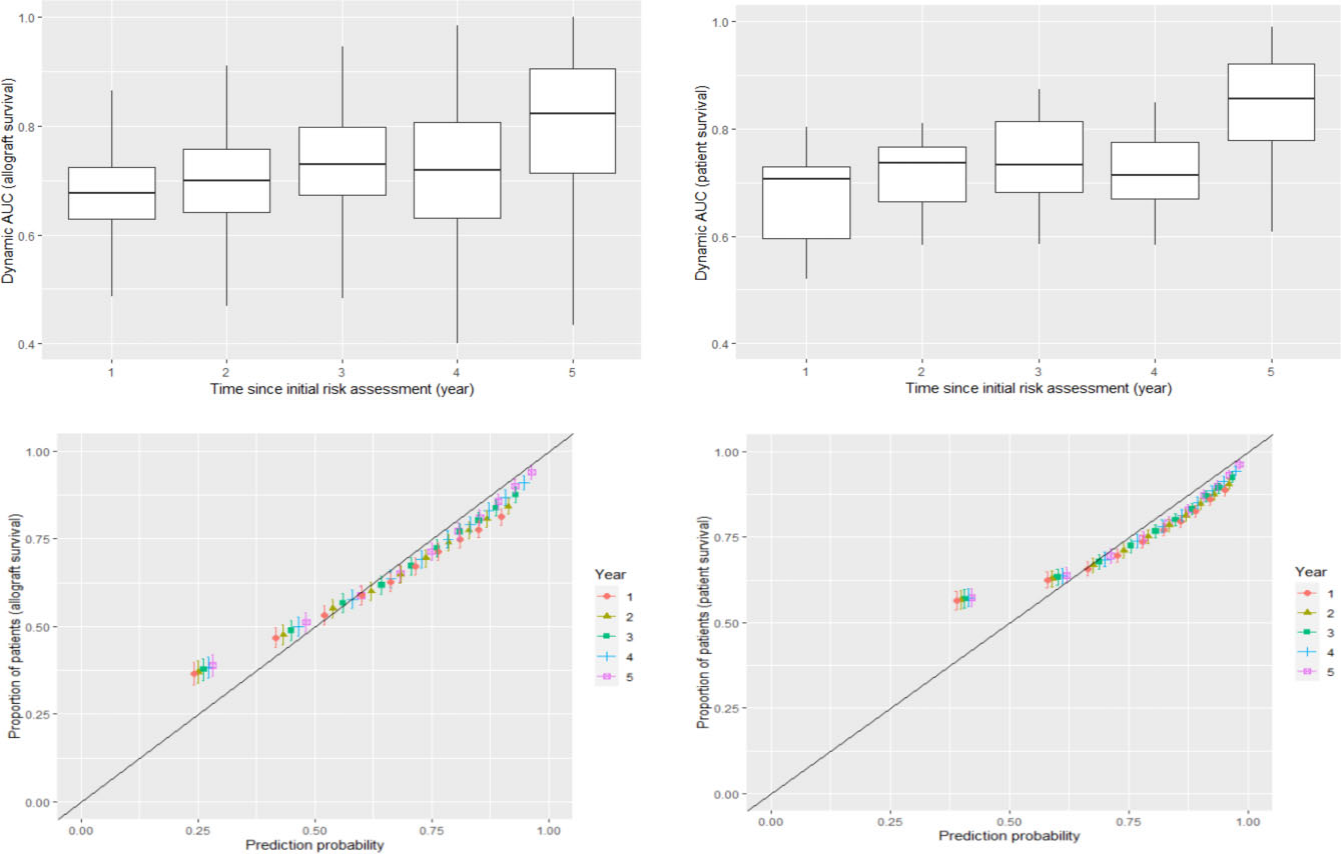
Performance characteristics of the dynamic based prediction model for kidney allograft and patient survival probabilities in the derivation cohort (Australia).

Figure 2 shows the boxplots of the dynamic AUC and the calibration plots over time for the derivation cohort. The median AUC between 0 and 5 years since the initial risk assessment varied between 0.70 and 0.83 for overall allograft survival probability, and between 0.73 and 0.89 for patient survival probability across the 5-year time-period. This suggested a high probability that the Bayesian joint model could accurately differentiate between transplant recipients who would or would not experience allograft loss or survival over the specified follow-up period of 5 years. Figure 2 also shows the calibration (i.e. the similarity between the predicted probabilities and the actual outcome) plots obtained from the model and also from the perfectly calibrated model using a diagonal line at the origin. This suggested that there was a strong agreement between the model-based prediction and actual allograft survival. The brier scores calculated from the model-based predictions were 0.083 for allograft and 0.057 for patient survival. Furthermore, the Spiegelhalter *Z*-test score provides a statistically significant calibration accuracy (*P* < .002) (Supplementary material).

### Performance characteristics of the dynamic joint model (validation cohort)

**Figure 3:**
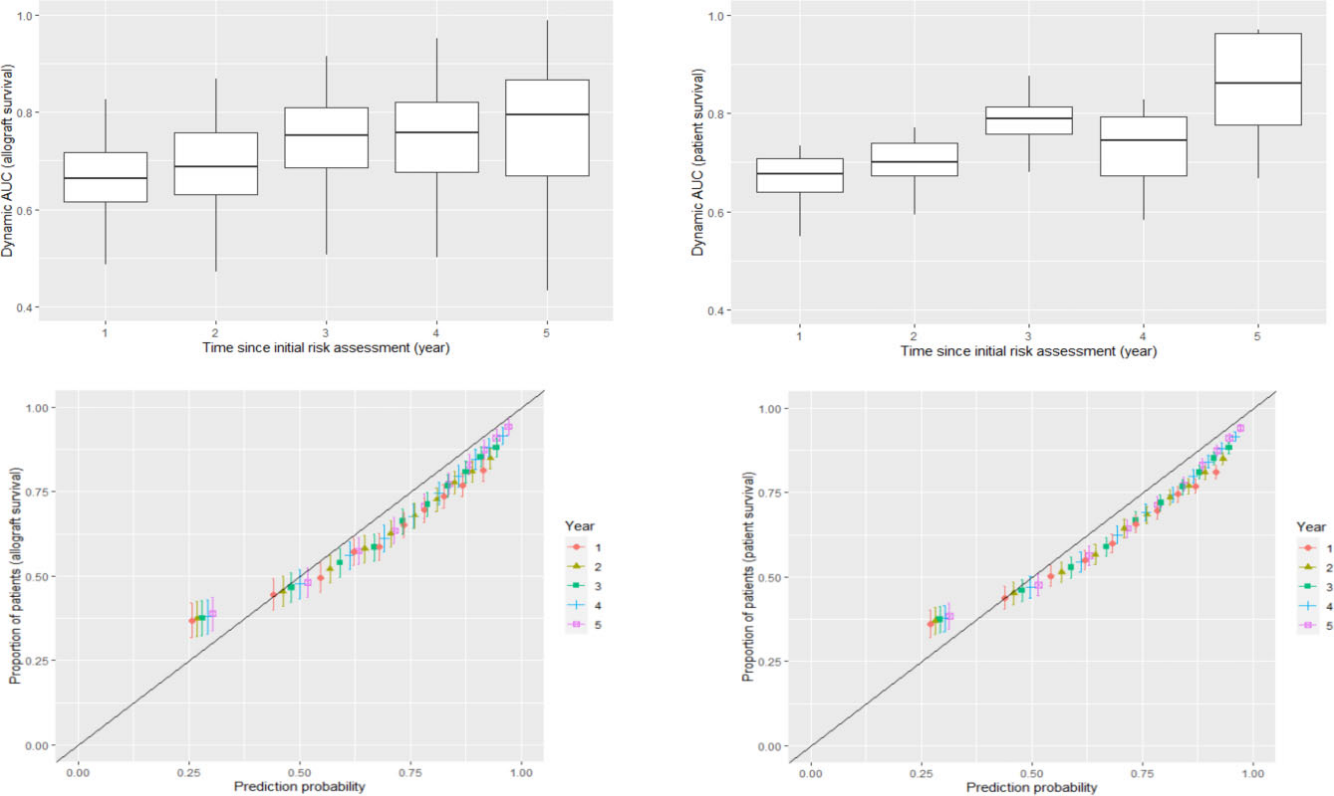
Performance characteristics of the dynamic based prediction model for kidney allograft and the patient survival probabilities in the validation cohort (New Zealand).

Figure 3 shows the boxplot of the estimated AUC measurements and the calibration plots for the validation cohort for average allograft survival time of up to 5 years. The median dynamic AUC of the joint model varied between 0.68 and 0.81 for the overall probability of survival of allograft, and the median dynamic AUC of the joint model for death varied between 0.67 and 0.87. This AUC shows good out-of-sample validation for the Bayesian joint model we developed for the allograft and patient survival. The calibration plots for the validation cohort also provided evidence of agreement between the actual observations and predictions. The smaller Brier scores (<0.1) and statistically significant (*P* < .004) Spiegelhalter *Z*-test indicated strong agreement between model-based predictions and actual observations at the validation cohort (Supplementary material).

### Dynamic prediction of allograft and patient survivals

**Figure 4:**
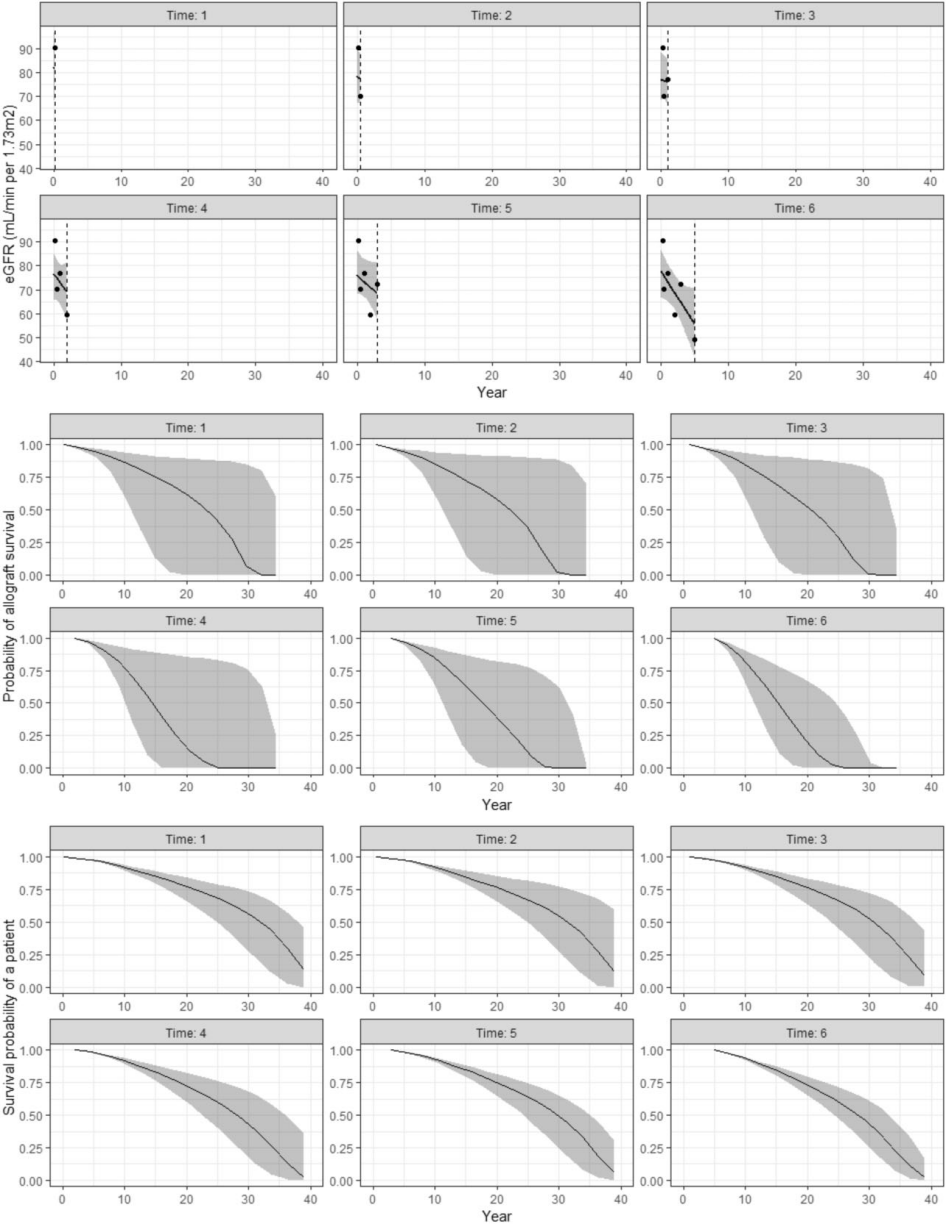
Dynamic prediction of kidney allograft and patient survivals using data from the validation cohort.

Figure 4 provides an illustrative example of the dynamic predictions of overall allograft and patient survival time using data from the external validation cohort. A total of six time-dependent measurements of eGFR and the relevant covariates (derived from the joint model) were observed. In this example, decreasing eGFR trajectory was predictive of both patient and overall allograft survivals. The prediction was performed dynamically, with an improvement in the predictive uncertainties over time when more information (with repeated eGFR measurements) was incorporated into the model. This is supported by smaller predictive uncertainty bands for both primary and secondary outcomes.

## DISCUSSION

Using data from this large cohort study comprising >14 000 primary kidney transplant recipients, we have developed a dynamic risk prediction model for overall allograft loss and death using eGFR trajectories. The time-dependent AUCs of the joint models were up to 0.81 for overall allograft loss and 0.87 for death, with improvements in predictive accuracy with increasing number of eGFR timepoints. The accuracies of the modeling were also confirmed in the validation cohort. Our model also showed strong agreement between the predicted and actual allograft survivals. Additionally, factors associated with eGFR decline over time were donor age, recipient age, and BMI. Previous dynamic modeling has used a combination of immunological, histological, clinical, and biochemical factors and found that the predictive performance of the final joint model was between 0.82 and 0.86 in external validated cohorts [11]. In this model, the minimal variables required in the model were repeated measures of eGFR and proteinuria. Furthermore, many of these variables, such as repeated donor-specific anti-HLA antibody (DSA) profiles and detailed biopsy data, are not always available in transplant units across all settings, particularly in low- to middle-income countries. Previous modeling also did not assess patient survival. Kidney function, allograft, and patient survival are considered the three most important outcome measures by patients, caregivers, and other stakeholders [6, 7, 24].

Serum creatinine measures are economical and readily available in all kidney transplant settings, even in low- and middle-income economies. This statistical model is not static but dynamic and is highly adaptive to changes by allowing the parameters to learn from new observations as they become available over time. However, implementing the joint model in clinical practice is not without challenges. The modeling is complex and may require longer computational time and costs. Furthermore, the model would require sufficient data for effective training, particularly for modeling long-term outcomes data in transplantation. This may pose potential barriers to implementation, as longer-term new and complete data may not be available across the exact time points for all participants in the context of observational studies. In addition, there is a strong assumption in the model that the eGFR trend is linear, but that may not necessarily be correct. In these situations, the longitudinal model can be extended based on a spline-based longitudinal model [25].

The large number of patients, the extended duration of follow-up, and the low proportion of patients with missing data, such as repeated eGFR measures, suggested that selection and selection biases were minimized. However, these strengths should be balanced against the limitations inherent in observational data. Validation of the model was performed in a cohort from New Zealand. Further confirmation of the model's findings in diverse global cohorts will improve the generalizability and robustness of the results. There are likely to be systematic differences in the clinical approach to patient management between transplant and non-transplant units. ANZDATA registry do not collect information on unmeasured confounders and mediators including the presence of pre-transplant or *de novo* DSA, extended allelic HLA-mismatches, repeated measures of proteinuria or albuminuria, medication non-adherence, therapeutic drug levels of calcineurin inhibitors, or changing immunosuppression regimens over time, and severity of prevalent vascular diseases, all of which could have potentially modified the association between eGFR trajectory and the study outcome measures. The positive change in mean eGFR over time associated with diabetic status may be counterintuitive. Such discrepancy may arise from the fact that diabetic status was only assessed at baseline (at the time of transplantation) and not repeatedly over time, leading to potential misclassification of the exposure, as the patients’ conditions may have evolved after baseline assessments. Also, the wide credible interval may suggest this finding is uncertain and should be interpreted with caution.

Although our prediction model did not include immunological and histological parameters, this is reflective of real-world clinical practice within the confines of data availability in lower to low-middle-income countries. Longitudinal long-term follow-up of kidney transplant recipients typically relies on repeated eGFR measures to establish allograft prognosis. An advantage of our dynamic prediction model is that it allows for a more accurate prediction of the risk of allograft loss and death at an individual level, which can be continuously refined with additional eGFR assessments. This can provide guidance to clinicians regarding the decision to undertake targeted investigations such as dnDSA monitoring or kidney allograft biopsy to direct clinical management among those at risk of early graft dysfunction. Furthermore, with the United States Food and Drug Administration accepting that eGFR decline as an acceptable clinical endpoint in clinical trials involving patients with chronic kidney disease [26, 27], triallists in kidney transplantation should consider a dynamic eGFR model-based approach in future trial design or interpretation of trial findings in kidney transplantation.

### Conclusions

We have developed and validated a dynamic eGFR risk prediction model to predict allograft loss and death in a large cohort of kidney transplant recipients in Australia and New Zealand. By incorporating a single metric in post-transplant care, which is readily accessible in most healthcare settings, including those in low-income countries, this user-friendly, dynamic bedside model can effectively facilitate the early identification of transplant recipients at risk of allograft loss and death. This tool enables timely intervention to prevent the progression of premature allograft loss and death, making it a valuable resource for improving patient outcomes across different clinical settings.

## Supplementary Material

sfae314_Supplemental_File

## Data Availability

The data used in this study can be requested from ANZDATA registry (email: requests@anzdata.org.au).
