# Supplementary material for: Dynamic prediction of kidney allograft and patient survival using post-transplant estimated glomerular filtration rate trajectory
Source: Clin Kidney J. 2024 Oct 16;17(11):sfae314. doi: 10.1093/ckj/sfae314 (PMC11551522; doi:10.1093/ckj/sfae314)
Supplement: sfae314_Supplemental_File [file sfae314_supplemental_file.pdf]

Supplementary Materials:

Dynamic prediction of kidney allograft and patient survival using post-transplant estimated glomerular filtration rate trajectory

Bakar K.S, Teixeira-Pinto A, Gately R, Boroumand F, Lim WH, Wong G

Dynamic Bayesian Joint Model for Allograft and Patient Survival using eGFR:

Let $y_i(t)$ be the eGFR measurements for ith individual/patient at time point t, where t can take maximum six time steps: 3, 6, 12-, 24-, 36- and 60-months post-transplant from the ANZDATA registry. Considering p number of covariates $\mathbf{x}_i'(t)$ and q dimensional design matrix $\mathbf{z}_i'(t)$, we write the linear-mixed model

$$y_i(t) = \mathbf{x}_i'(t)\boldsymbol{\beta} + \mathbf{z}_i'(t)\mathbf{b}_i + \epsilon_i(t)$$

Where, $\boldsymbol{\beta} = \left(\beta_1, \dots \beta_p\right)'$is the model coefficient, $\mathbf{b}_i = \left(b_{1i}, \dots, b_{qi}\right)'$ is the random effects and $\epsilon_i(t)$ is the white noise. In this paper, we developed model using random intercept for patient i and corresponding slopes for the longitudinal time-series. Considering mean process with linear predictor $\eta_i(t)$, we write

$$g^{-1}\left(\eta_i(t)\right) = g^{-1}(\mathbf{x}_i'(t)\boldsymbol{\beta} + \mathbf{z}_i'(t)\mathbf{b}_i)$$

Now, for defining time-to-event model for allograft and patient survival, let us consider the allograft event time for individual i as $T_i^{\mathrm{g}} = \min\{T_i^{\mathrm{g}*}, C_i^{\mathrm{g}}\}$ and for the ith patient survival $T_i^{\mathrm{p}} = \min\{T_i^{\mathrm{p}*}, C_i^{\mathrm{p}}\}$, where $T_i^{\mathrm{g}*}$, $T_i^{\mathrm{p}*}$ and C_i^{g}, C_i^{p} are the true event and censoring time for allograft and patient survival respectively. Thus, we write the survival models:

$$h_i^{\mathrm{g}}(t) = h_0^{\mathrm{g}}(t)\exp\left(\boldsymbol{x}_i'(t)\widetilde{\boldsymbol{\beta}}^{\mathrm{g}} + \mathrm{w}_i(t)\right)$$
$$h_i^{\mathrm{p}}(t) = h_0^{\mathrm{p}}(t)\exp\left(\boldsymbol{x}_i'(t)\widetilde{\boldsymbol{\beta}}^{\mathrm{p}} + \mathrm{w}_i(t)\right)$$

Where, $\widetilde{\boldsymbol{\beta}}^{\mathrm{g}} = \left(\beta_1^{\mathrm{g}}, \dots \beta_p^{\mathrm{g}}\right)'$ and $\widetilde{\boldsymbol{\beta}}^{\mathrm{p}} = \left(\beta_1^{\mathrm{p}}, \dots \beta_p^{\mathrm{p}}\right)'$ are the parameters for the allograft and patient survival models and $\mathrm{w}_i(t)$ is the latent process that captures the association structure between the eGFR and allograft and patient survival event processes. We use a simple structure for the association structure, where we define $\mathrm{w}_i(t) = \alpha \times \eta_i(t)$ and α is known as the association parameter. Hence, we write the allograft and patient survival probabilities of individual i as:

$$\mathrm{S}_i^{\mathrm{g}}(t) = \Pr\left(T_i^{\mathrm{g}*} \geq t\right) = \exp\left(-\int h_i^{\mathrm{g}}(t)dt\right)$$
$$\mathrm{S}_i^{\mathrm{p}}(t) = \Pr\left(T_i^{\mathrm{p}*} \geq t\right) = \exp\left(-\int h_i^{\mathrm{p}}(t)dt\right)$$

We use normal prior distribution for model parameters $\boldsymbol{\beta}$, $\widetilde{\boldsymbol{\beta}}^{\mathrm{g}}$ and $\widetilde{\boldsymbol{\beta}}^{\mathrm{p}}$ with mean zero and wide variance to maintain non-informativeness of the prior distribution. Let, $\boldsymbol{\theta}$ contains all model parameters and unknowns, and hence, the posterior distribution can be written as:

$$\pi(\boldsymbol{\theta}, \mathbf{b}|\mathbf{y}, T^{\mathrm{g}}, T^{\mathrm{p}})\ \alpha \prod \pi(\mathbf{y}|\boldsymbol{\theta}, \mathbf{b})\,\pi(T^{\mathrm{g}}|\boldsymbol{\theta}, \mathbf{b})\pi(T^{\mathrm{p}}|\boldsymbol{\theta}, \mathbf{b})\pi(\mathbf{b}|\boldsymbol{\theta})\pi(\boldsymbol{\theta})$$

Where, $\pi(\boldsymbol{\theta})$ is the prior distributions of the model parameters. For model predictions, we use individual specific approach. Let $\mathbf{y}_i^*$ be the eGFR prediction and $\mathrm{S}_i^{\mathrm{g}*}(t)$ & $\mathrm{S}_i^{\mathrm{p}*}$ be the predicted survival probability at time t for the $i$the individual. Hence, we solve the following equations of the posterior predictive distributions to get the predictions:

$$\pi(\mathbf{y}_i^*|\mathbf{M}) = \iint \pi(\mathbf{y}_i^*|\boldsymbol{\theta}, \mathbf{b})\pi(\boldsymbol{\theta}, \mathbf{b}|\mathbf{M})\, d\mathbf{b}\, d\boldsymbol{\theta}$$

$$\pi\left(\mathrm{S}_i^{\mathrm{g}*}(t)|\mathbf{M}\right) = \iint \pi\left(\mathrm{S}_i^{\mathrm{g}*}(t)|\boldsymbol{\theta}, \mathbf{b}\right)\pi(\boldsymbol{\theta}, \mathbf{b}|\mathbf{M})\, d\mathbf{b}\, d\boldsymbol{\theta}$$

$$\pi\left(\mathrm{S}_i^{\mathrm{p}*}(t)\middle|\mathbf{M}\right) = \iint \pi\left(\mathrm{S}_i^{\mathrm{p}*}(t)\middle|\boldsymbol{\theta},\mathbf{b}\right)\pi(\boldsymbol{\theta},\mathbf{b}|\mathbf{M})\, d\mathbf{b}\, d\boldsymbol{\theta}$$

Where, $\mathbf{M}$ is the collection of outcome/data from the model.

Prior Sensitivity:

Prior distributions		Log of the posterior predictive density
Keeping other parameters non-informative, i.e., wide prior variance		
$\boldsymbol{\beta} \sim \boldsymbol{N}(\mathbf{0},\Sigma_\beta)$ $\Sigma_\beta = \begin{pmatrix} \sigma_{1\beta}^2 & \cdots & 0 \\ \vdots & \ddots & \vdots \\ 0 & \cdots & \sigma_{p\beta}^2 \end{pmatrix}$	$(\sigma_{1\beta}^2, \dots \sigma_{p\beta}^2) = 10^4$	- 408981.28
	$(\sigma_{1\beta}^2, \dots \sigma_{p\beta}^2) = 10$	- 409302.11
	$(\sigma_{1\beta}^2, \dots \sigma_{p\beta}^2) = 1$	- 409347.69
Keeping other parameters non-informative, i.e., wide prior variance		
$\widetilde{\boldsymbol{\beta}}^{\mathrm{g}} \sim \boldsymbol{N}(\mathbf{0},\Sigma_{\mathrm{g}})$ $\Sigma_{\mathrm{g}} = \begin{pmatrix} \sigma_{1\mathrm{g}}^2 & \cdots & 0 \\ \vdots & \ddots & \vdots \\ 0 & \cdots & \sigma_{p\mathrm{g}}^2 \end{pmatrix}$	$(\sigma_{1\mathrm{g}}^2, \dots \sigma_{p\mathrm{g}}^2) = 10^4$	- 408981.28
	$(\sigma_{1\mathrm{g}}^2, \dots \sigma_{p\mathrm{g}}^2) = 10$	- 409530.12
	$(\sigma_{1\mathrm{g}}^2, \dots \sigma_{p\mathrm{g}}^2) = 1$	- 409561.76
Keeping other parameters non-informative, i.e., wide prior variance		
$\widetilde{\boldsymbol{\beta}}^{\mathrm{p}} \sim \boldsymbol{N}(\mathbf{0},\Sigma_{\mathrm{p}})$ $\Sigma_{\mathrm{p}} = \begin{pmatrix} \sigma_{1\mathrm{g}}^2 & \cdots & 0 \\ \vdots & \ddots & \vdots \\ 0 & \cdots & \sigma_{p\mathrm{p}}^2 \end{pmatrix}$	$(\sigma_{1\mathrm{p}}^2, \dots \sigma_{p\mathrm{p}}^2) = 10^4$	- 408981.28
	$(\sigma_{1\mathrm{p}}^2, \dots \sigma_{p\mathrm{p}}^2) = 10$	- 409127.05
	$(\sigma_{1\mathrm{p}}^2, \dots \sigma_{p\mathrm{p}}^2) = 1$	- 409122.52

***Descriptive Results*:**

Bivariate Analyses (eGFR and background/clinical variables for both derivation and validation cohorts – combined Australian & New Zealand Data):

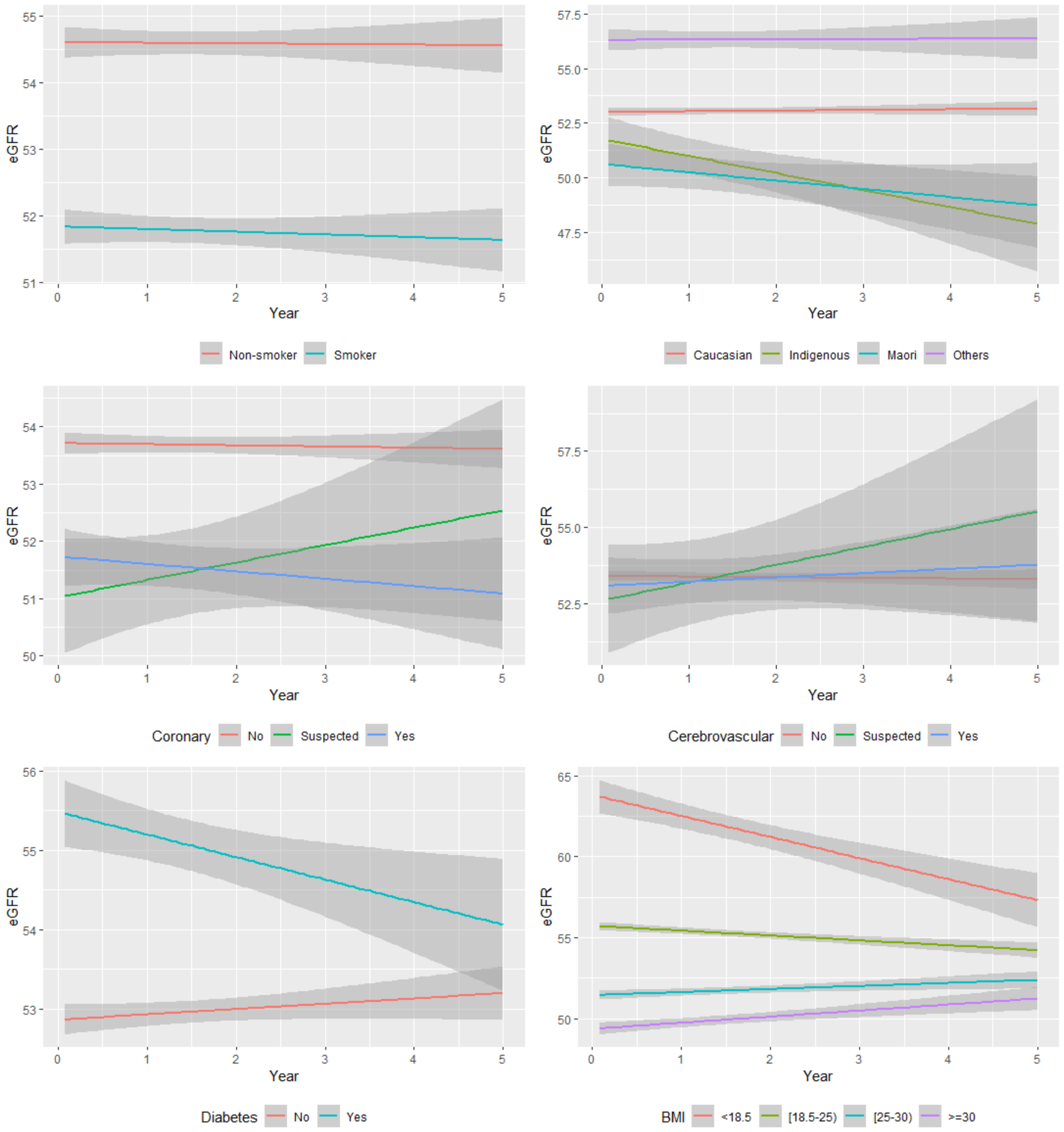

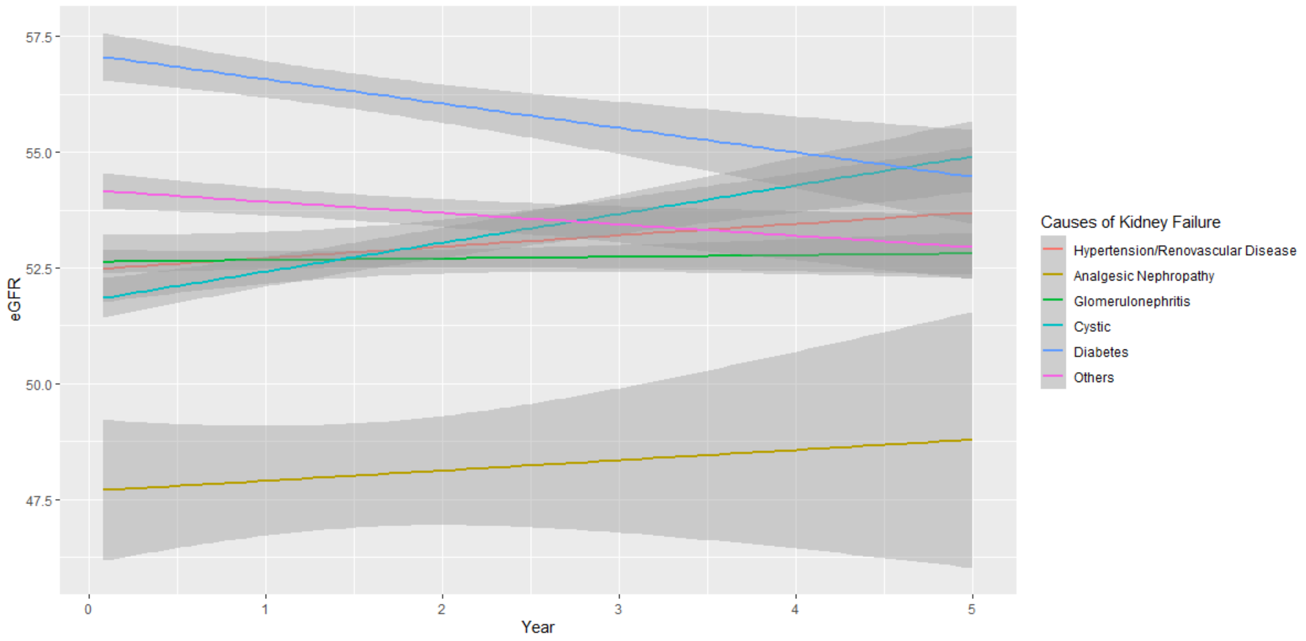

Calibration statistics:

Overall risk assessment	Brier score	Average absolute difference in predicted and loess-calibrated probabilities	Spiegelhalter Z-test score for calibration accuracy (p-value)
Kidney allograft survival			
Derivation cohort (AUS)	0.083	0.192	-3.343 (0.001)
Validation cohort (NZ)	0.085	0.179	-2.913 (0.004)
Patient survival			
Derivation cohort (AUS)	0.057	0.170	-3.055 (0.002)
Validation cohort (NZ)	0.077	0.176	-3.201 (0.001)

Linear vs. Non-linear Mixed-Models:

We checked the linearity assumptions of the mixed model used for the longitudinal eGFR data. The Q-Q (quantile-quantile) probability plots show an approximately linear shape (see the plots below). Furthermore, we also compared the linear mixed-model with a non-linear (spline-based) mixed model, where we used b-spline for the time-segments with optimized knots (implemented through R package gamm4). We find that the Bayesian Informatic Criterion, i.e., BIC (we also checked for AIC) is smaller (BIC=708692.5, AIC=708372.5) for the linear model compared to the BIC for the spline model (BIC=769516.6, AIC=769214.8). This reflects a good model fit for the linear mixed-model compared to the non-linear model. This also reflects that the penalty for the spline model is much higher compared to the linear model due to the consideration of increase in model parameters. Note that, we only implemented the mixed-model in this context to understand the model performance based on linearity and non-linearity. Furthermore, the dynamic nature of the Bayesian joint model for each time point considered as a latent process $[w_i(t) = \alpha \times \eta_i(t)]$ of eGFR in the survival model, where the latent process was used to adjust the eGFR predictions and hence predictions for the allograft and patient's survival probabilities.

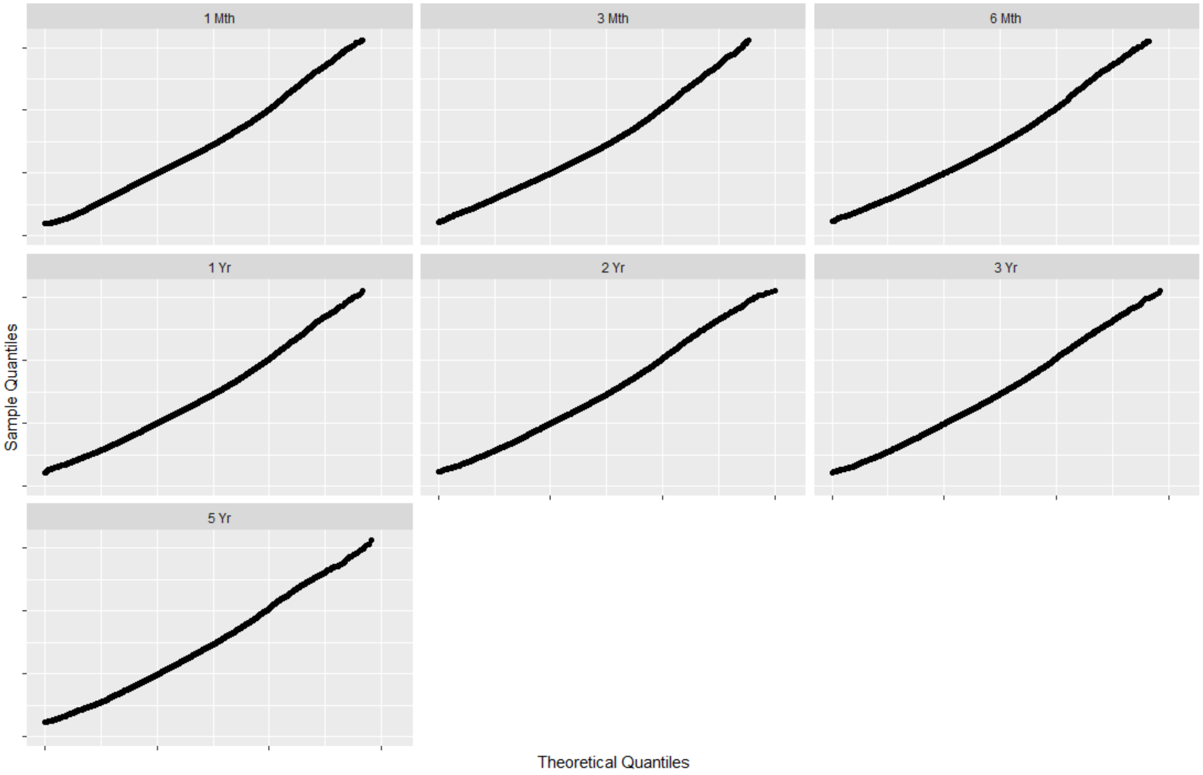

Calibration plots:

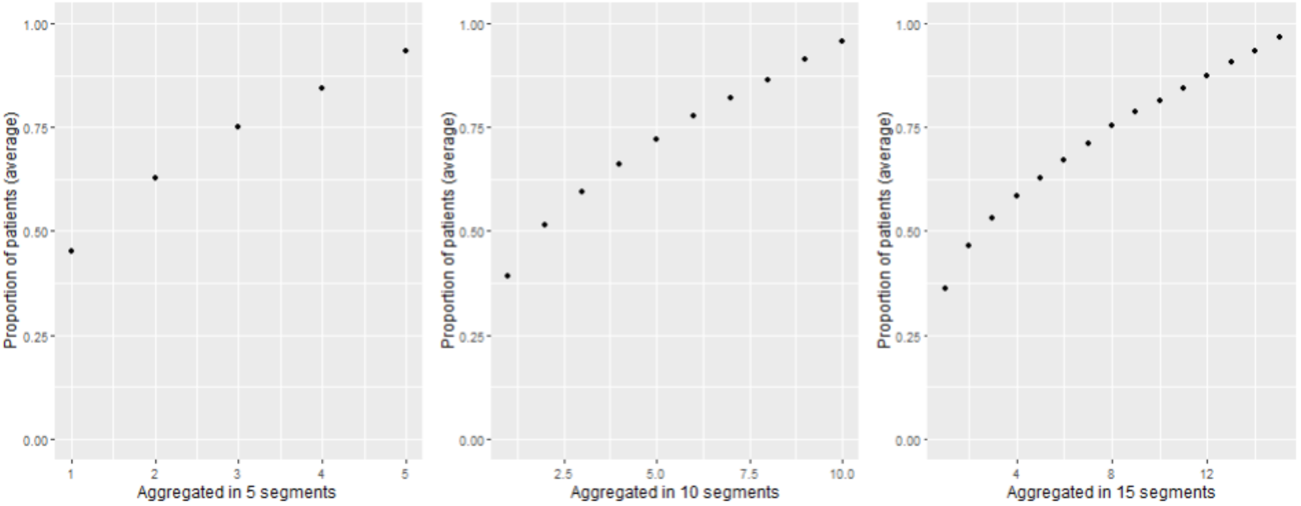

Figure showing the average observed proportion of patients plots by different segments (first plot with 5 segments, second plot with 10 segments and last plot with 15 segments).

Shiny Application:

App link: https://centreforkidneyresearch.shinyapps.io/CKR_JMapp/
Github code link: https://github.com/ksbakar/BayesJointModelling/tree/main/CKR_JMapp

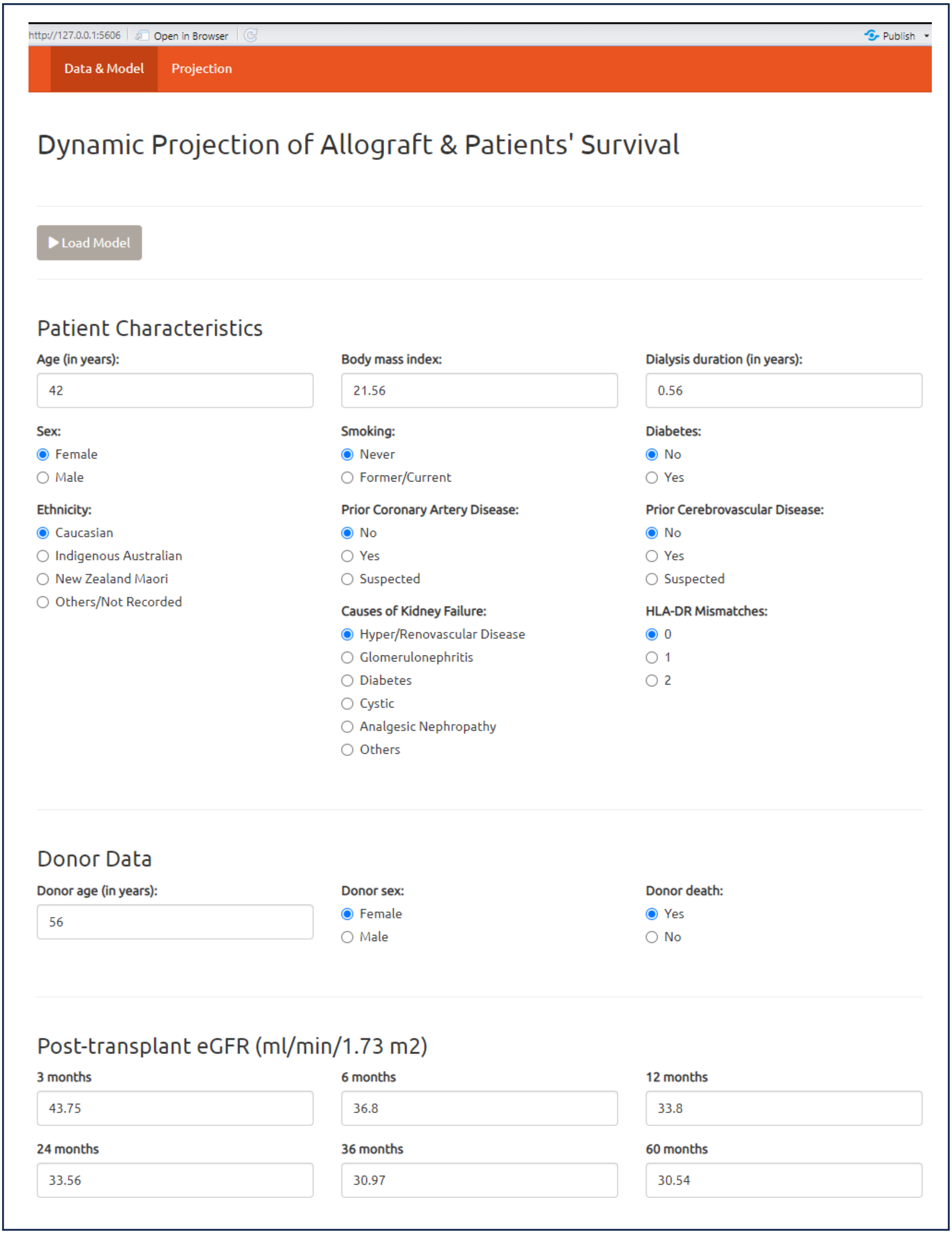
http://127.0.0.1:5606
Open in Browser
Publish
Data & Model
Projection
Dynamic Projection of Allograft & Patients' Survival
Load Model
Patient Characteristics
Age (in years):
42
Body mass index:
21.56
Dialysis duration (in years):
0.56
Sex:
Female
Male
Smoking:
Never
Former/Current
Diabetes:
No
Yes
Ethnicity:
Caucasian
Indigenous Australian
New Zealand Maori
Others/Not Recorded
Prior Coronary Artery Disease:
No
Yes
Suspected
Prior Cerebrovascular Disease:
No
Yes
Suspected
Causes of Kidney Failure:
Hyper/Renovascular Disease
Glomerulonephritis
Diabetes
Cystic
Analgesic Nephropathy
Others
HLA-DR Mismatches:
0
1
2
Donor Data
Donor age (in years):
56
Donor sex:
Female
Male
Donor death:
Yes
No
Post-transplant eGFR (ml/min/1.73 m2)
3 months
43.75
6 months
36.8
12 months
33.8
24 months
33.56
36 months
30.97
60 months
30.54

Outcome:

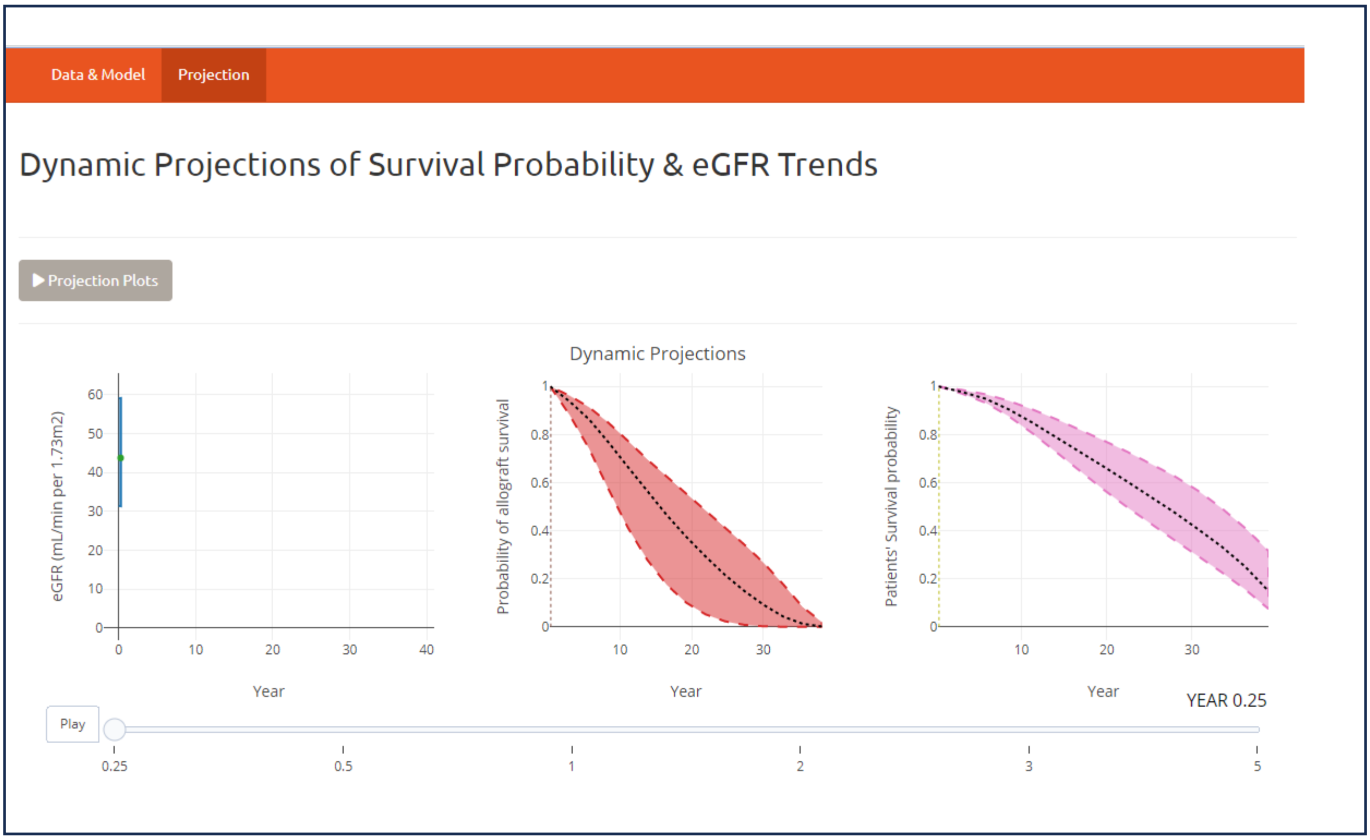
Data & Model
Projection
Dynamic Projections of Survival Probability & eGFR Trends
Projection Plots
Dynamic Projections
eGFR (mL/min per 1.73m2)
Year
Probability of allograft survival
Year
Patients' Survival probability
Year
YEAR 0.25
Play
0.25
0.5
1
2
3
5

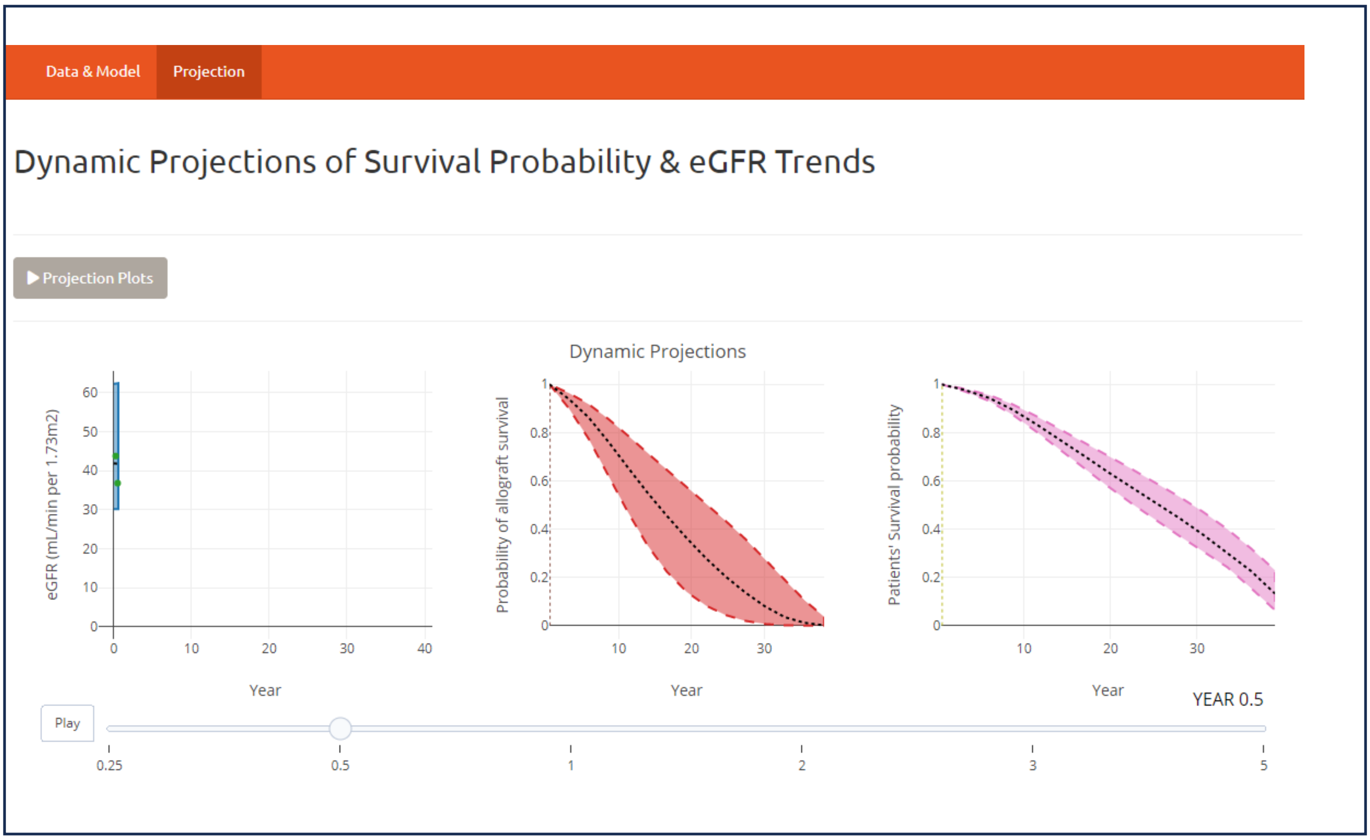
Data & Model
Projection
Dynamic Projections of Survival Probability & eGFR Trends
Projection Plots
Dynamic Projections
eGFR (mL/min per 1.73m2)
Year
Probability of allograft survival
Year
Patients' Survival probability
Year
YEAR 0.5
Play
0.25
0.5
1
2
3
5

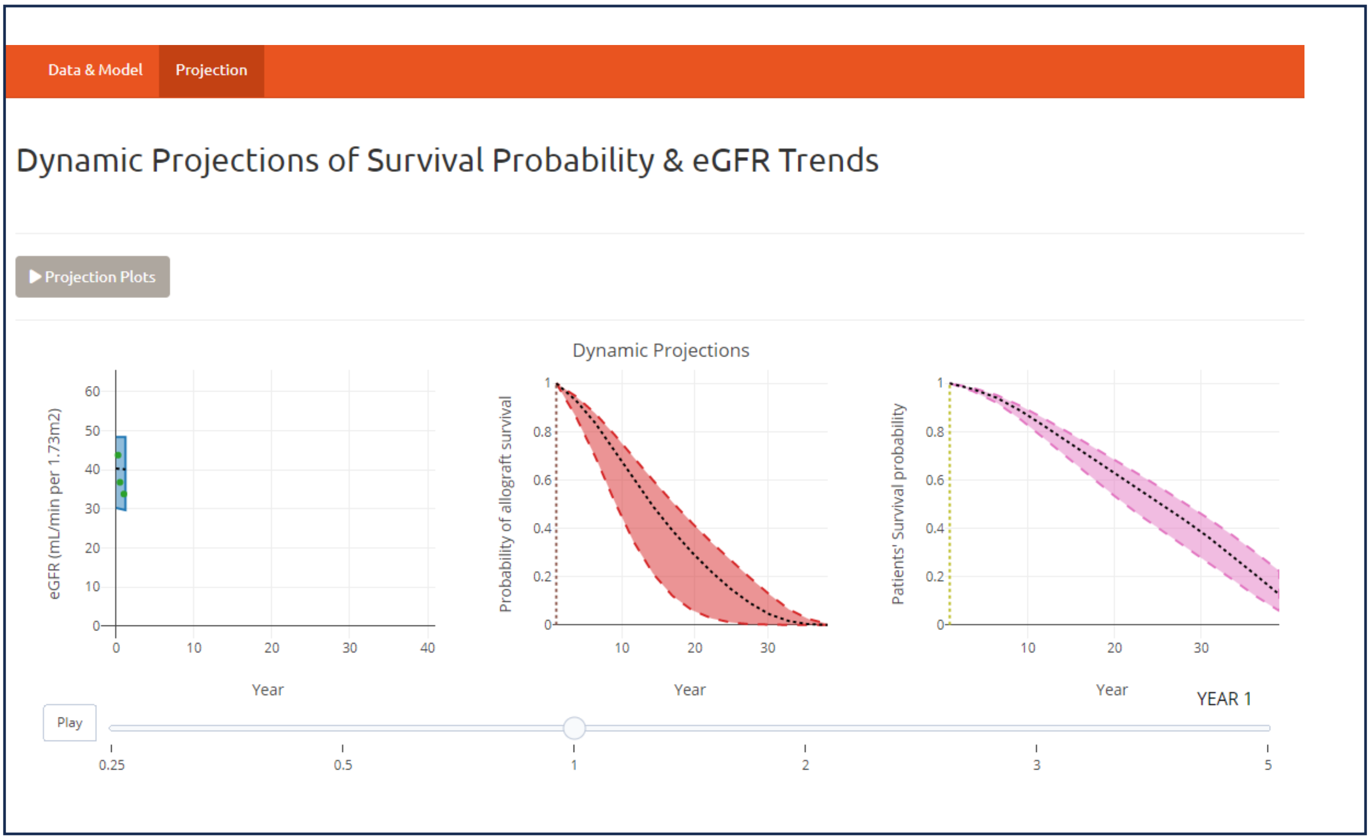
Data & Model
Projection
Dynamic Projections of Survival Probability & eGFR Trends
Projection Plots
Dynamic Projections
eGFR (mL/min per 1.73m2)
Probability of allograft survival
Patients' Survival probability
Year
Year
Year
YEAR 1
Play
0.25
0.5
1
2
3
5

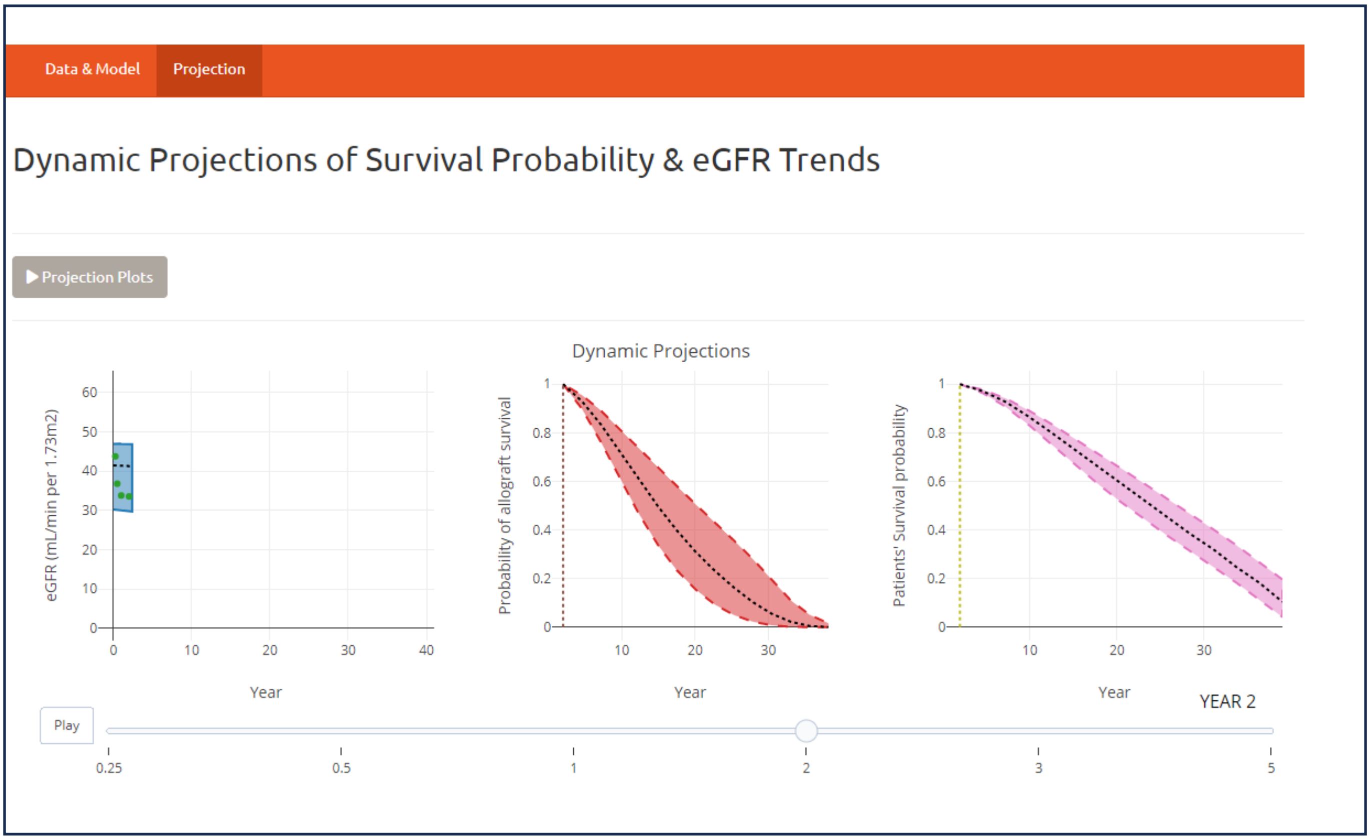
Data & Model
Projection
Dynamic Projections of Survival Probability & eGFR Trends
Projection Plots
Dynamic Projections
eGFR (mL/min per 1.73m2)
Probability of allograft survival
Patients' Survival probability
Year
Year
Year
YEAR 2
Play
0.25
0.5
1
2
3
5

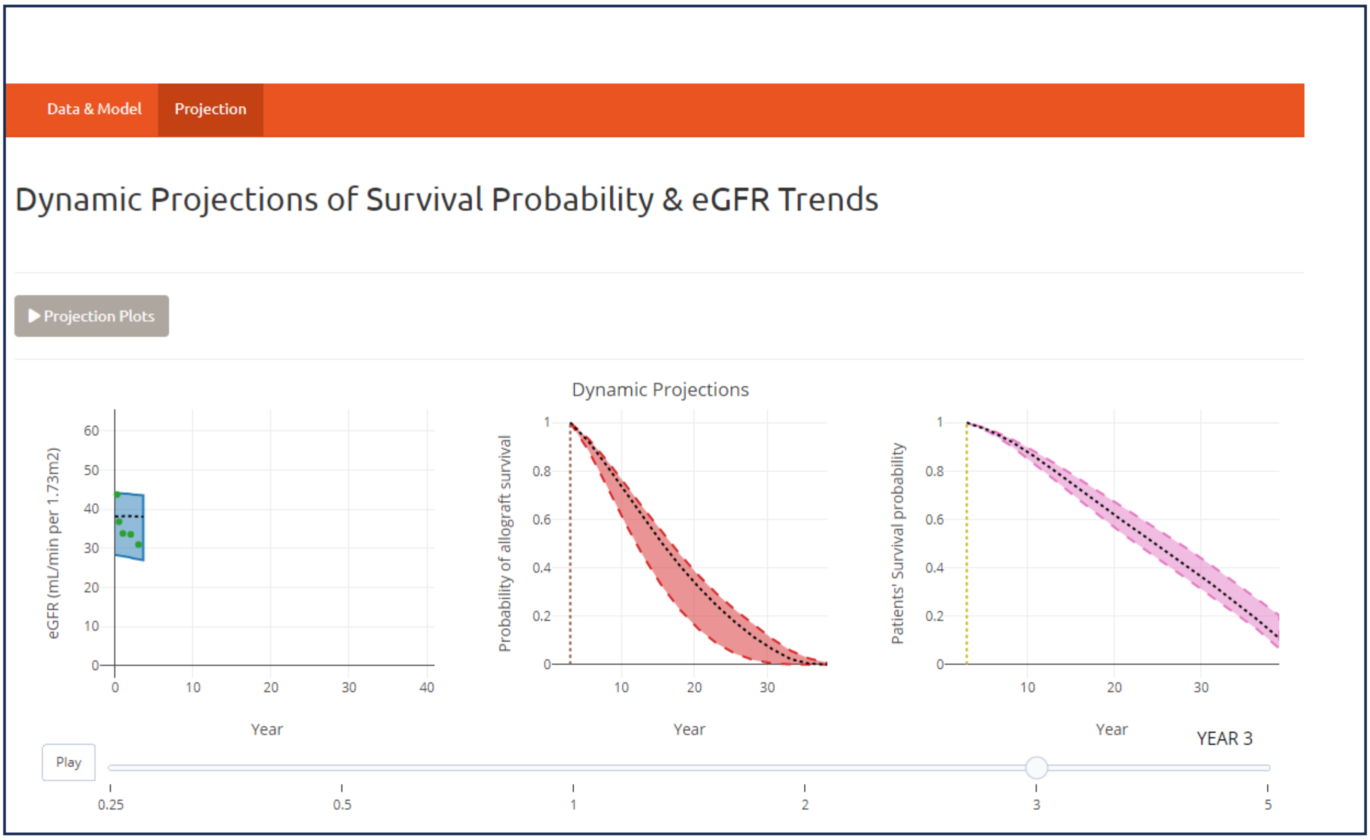
Data & Model
Projection
Dynamic Projections of Survival Probability & eGFR Trends
▶ Projection Plots
Dynamic Projections
eGFR (mL/min per 1.73m2)
Probability of allograft survival
Patients' Survival probability
Year
Year
Year
YEAR 3
Play
0.25
0.5
1
2
3
5

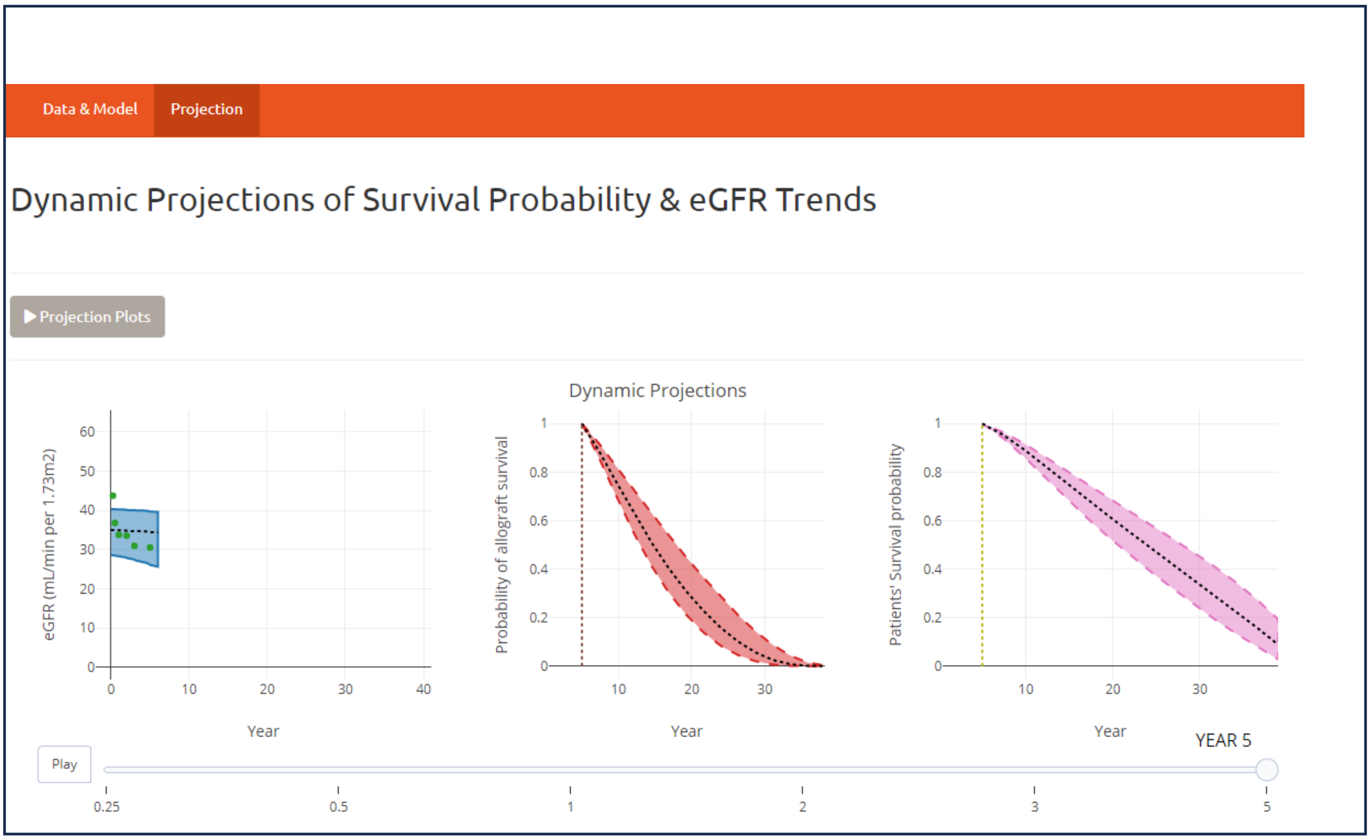
Data & Model
Projection
Dynamic Projections of Survival Probability & eGFR Trends
▶ Projection Plots
Dynamic Projections
eGFR (mL/min per 1.73m2)
Probability of allograft survival
Patients' Survival probability
Year
Year
Year
YEAR 5
Play
0.25
0.5
1
2
3
5
